# Supplementary material for: Complex‐centric proteome profiling by SEC‐SWATH‐MS
Source: Mol Syst Biol. 2019 Jan 14;15(1):e8438. doi: 10.15252/msb.20188438 (PMC6346213; doi:10.15252/msb.20188438)
Supplement: Supplementary file 8 — Dataset EV7 [file MSB-15-e8438-s008.zip › feature_plots_string/Q96EB1.pdf]

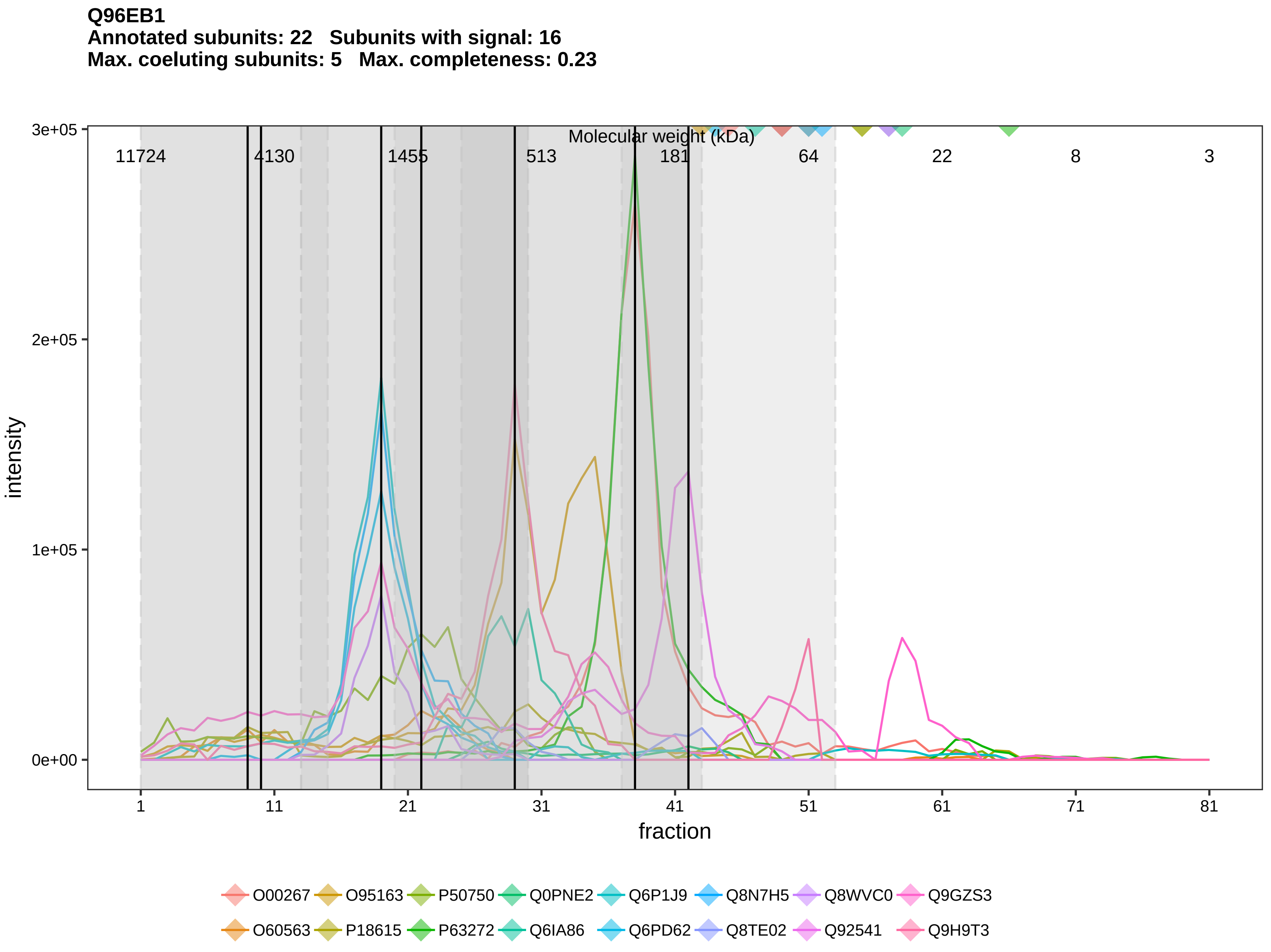

Q96EB1
Annotated subunits: 22 Subunits with signal: 16
Max. coeluting subunits: 5 Max. completeness: 0.23
Molecular weight (kDa)
11724
4130
1455
513
181
64
22
8
3
intensity
3e+05
2e+05
1e+05
0e+00
fraction
1
11
21
31
41
51
61
71
81
O00267
O95163
P50750
Q0PNE2
Q6P1J9
Q8N7H5
Q8WVC0
Q9GZS3
O60563
P18615
P63272
Q6IA86
Q6PD62
Q8TE02
Q92541
Q9H9T3
